# Passive vs Active Nighttime Smartphone Use as Markers of Next-Day Suicide Risk

**DOI:** 10.1001/jamanetworkopen.2025.42675

**Published:** 2025-11-11

**Authors:** Ross Jacobucci, Stephanie G. Jones, Miguel Blacutt, Brooke A. Ammerman

**Affiliations:** 1 Center for Healthy Minds, University of Wisconsin-Madison; 2 Department of Psychiatry, University of Wisconsin-Madison; 3 Wisconsin Institute for Sleep and Consciousness, University of Wisconsin-Madison; 4 Department of Psychology, University of Notre Dame, South Bend, Indiana; 5 Department of Psychology, University of Wisconsin-Madison

## Abstract

**Importance:**

Nighttime represents a high-risk period for suicidal thoughts, yet current understanding of how digital technology use during these hours influences suicide risk remains limited. Distinguishing between passive consumption and active engagement may inform the future development of targeted suicide intervention efforts.

**Objective:**

To examine whether the timing and type (passive vs active) of nighttime smartphone use are associated with next-day suicidal ideation and suicide planning in adults at a high risk of suicide.

**Design, Setting, and Participants:**

This survey and passive data sensing study used ecological momentary assessment (EMA) and smartphone screenshots collected every 5 seconds during active phone use over 28 days per participant. Data were collected from August 24, 2022, to January 9, 2024. The community-based sample included adults aged 18 years or older with past-month suicidal thoughts or behaviors from South Bend, Indiana, and the surrounding area. Inclusion criteria were smartphone ownership and variability in key variables across the 28-day period.

**Exposure:**

Timing and type of nighttime smartphone use were assessed in 3 ways: (1) maximum phone-free interval (8:00 pm to 10:00 am), (2) use during self-reported sleep windows, and (3) hourly use (11:00 pm to 8:00 am). Keyboard presence, detected via a deep learning model trained on 1000 manually annotated screenshots, served as a proxy for active engagement.

**Main Outcomes and Measures:**

The primary outcomes were next-day passive suicidal ideation, active suicidal ideation, and suicide planning assessed via EMA (6 prompts daily). Bayesian multilevel models tested within-person and between-person associations.

**Results:**

The study enrolled 79 participants (mean [SD] age, 35.2 [11.1] years; 54 female [68.3%]). The overall EMA compliance rate was 68.8%, and participants generated 7.5 million screenshots (of which 1.05 million [14.0%] showed keyboard presence). Late-night (11:00 pm to 1:00 am) phone use was consistently associated with higher next-day passive or active suicidal ideation and suicide planning. Participants with 7- to 9-hour phone-free gaps showed the lowest suicidal ideation compared with those with 4- to 7-hour phone gaps (B = 0.35; 95% credible interval [CrI], 0.14-0.55). Keyboard activity was associated with lower suicidal ideation when occurring during middle-night hours (1:00 to 5:00 am) (simple slope, −0.10; 95% CrI, −0.13 to −0.07), self-reported sleep windows for both passive (B = −0.14; 95% CrI, −0.24 to −0.02) and active (B = −0.13; 95% CrI, −0.24 to −0.02) suicidal ideation, and nonsleep periods for both passive (B = −0.16; 95% CrI, −0.29 to −0.03) and active (B = −0.12; 95% CrI = −0.24 to −0.001) suicidal ideation. These timing-dependent, within-person associations were independent of total usage.

**Conclusions and Relevance:**

This study found temporally specific associations between nighttime smartphone use and next-day suicidal ideation and suicide planning. Late-night use signaled vulnerability, while middle-night active engagement seemed protective. These findings differentiate harmful passive consumption from potentially adaptive digital coping, potentially serving to advance precision approaches to suicide prevention.

## Key Points

**Question:**

Are the timing and type (passive vs active) of nighttime smartphone use associated with next-day suicidal ideation and suicide planning in high-risk adults?

**Findings:**

In this study combining surveys and passive data collection in 79 adults with recent suicidal thoughts, late-night phone use (11:00 pm to 1:00 am) was associated with higher next-day suicide risk, while keyboard-based activity during middle-night hours (1:00 to 5:00 am) was associated with lower risk.

**Meaning:**

These findings suggest that specific usage patterns of nighttime smartphone use may be a more important focus for future suicide prevention interventions than total screen time.

## Teaser

This survey study examines whether the timing and type (passive vs active) of nighttime smartphone use are associated with next-day suicidal ideation and suicide planning among adults at high risk of suicide.

## Introduction

Suicide remains a major public health crisis, with more than 49 000 deaths reported in the US in 2022, an increase of 2.6% from the prior year, and more than 10 million adults experiencing suicidal thoughts annually.^1,2^ To reduce this burden, it is critical to identify proximal, modifiable risk factors that can inform real-time interventions. Recent work has identified the nighttime period as a potential high-risk window, with sleep disturbances, such as increased latency, poor continuity, and shortened duration, being associated with next-day increases in suicidal ideation.^3,4,5^ Yet, relatively little is known about modifiable nighttime behaviors that may be associated with disrupted sleep and heighten suicide risk.

One increasingly common behavior is nighttime smartphone use, which can interfere with sleep through multiple mechanisms, including blue light exposure, emotionally arousing content, and disruptive notifications.^6,7^ With more than 96% of adults in the US using smartphones,^8^ such use is now embedded in daily life. Cross-sectional studies have suggested that phone use before bed, nighttime texting, and being awakened by notifications are associated with poorer sleep outcomes and higher psychological distress.^9,10,11^ Notably, individuals experiencing suicidal thoughts often use their phones late at night,^12^ and self-reported nighttime use has been associated with suicidal ideation.^13^ In parallel, digital behavior tracking has shown that nighttime phone activity, including calls, texts, and social media use, is associated with elevated stress and depressive symptoms.^14^ Despite these associations, no studies have tested whether objectively measured nighttime smartphone use is associated with next-day suicide risk.

Compounding this gap is that not all smartphone use is the same. A growing body of evidence distinguishes between passive use (eg, scrolling) and active use (eg, messaging, posting), with the former associated with negative affect and social disconnection and the latter more often with positive emotional outcomes.^15,16^ Preliminary evidence has suggested that these distinctions may matter for suicide risk. For example, Dissing et al^14^ found that certain patterns of active use at night are associated with lower emotional distress. However, few studies have incorporated these behavioral distinctions, and none have done so using high-frequency, passively collected data.

In this study, we use Screenomics,^17^ a digital phenotyping approach that captures smartphone screenshots every 5 seconds during phone use, alongside ecological momentary assessment (EMA) to examine whether nighttime smartphone use is associated with next-day suicidal ideation and suicide planning in a high-risk adult sample. We operationalized nighttime phone use across multiple levels of specificity to test whether the timing and type of use (ie, active vs passive engagement) are differentially associated with next-day suicide risk. This approach addresses major limitations of prior research, including reliance on self-report, low temporal resolution, and failure to differentiate meaningful behavioral subtypes. Our study aims to inform the identification of high-risk periods and guide the development of just-in-time digital interventions for suicide prevention.

## Methods

This survey and passive data sensing study and all its procedures were approved by the institutional review boards of the University of Notre Dame and University of Wisconsin-Madison. All participants provided written inform consent after having been briefed on data collection, security, and confidentiality and discussion regarding limits of confidentiality and mandatory risk procedures. The report adheres to the Association of Public Opinion and Survey Research Professionals (AAPOR) reporting guideline for survey studies.

### Participants

Adults aged 18 years or older were recruited via online advertisements, community flyers, and local community mental health centers in South Bend, Indiana, and the surrounding area. Eligibility criteria included past-month suicidal thoughts or behaviors, Android smartphone (Android Inc) ownership (for screenshot capture), and variability in key variables (eg, suicidal ideation, image count) across a 28-day period. Trained study team members collected all demographic data via predefined categories, including self-reported race (American Indian or Alaska Native, Black or African American, White, multiracial, not listed) and ethnicity (Hispanic/Latino, non-Hispanic/Latino) for the purposes of characterizing the sample and the ability to consider individual differences as meaningful covariates.

### Procedures

Participants completed a laboratory visit with diagnostic interview (Mini-International Neuropsychiatric Interview^18^) and a suicide risk interview (Self-Injurious Thoughts and Behaviors Interview^19^), which were used to both describe the sample and confirm eligibility criteria. Participants also received instructions for the 28-day EMA period (6 prompts per day, randomized into 2-hour windows across a 12-hour period) and the Screenomics protocol,^17^ in which screenshots were captured at 5-second intervals during phone use via ScreenLife Capture.^20^ Screenshots were stored locally, encrypted, and transmitted in bundles to secure research servers. Participants who reported imminent suicide risk were contacted by the study team. Participants were renumerated up to $240 for participation. Data collection occurred from August 24, 2022, to January 9, 2024.

### Measures

Momentary suicidal ideation (ie, queried as “At this moment…”) was assessed using 4 EMA items, including 2 items for passive suicidal ideation (ie, “Life is not worth living for me.” “There are more reasons to die than to live for me.”) and 2 items for active suicidal ideation (ie, “I think about taking my life.” “I want to die.”),^21^ scored on a 5-point Likert scale (from 1 [not at all] to 5 [very much]) and summed to create 2 composite scores. Suicide planning was measured using 3 adapted items from the Beck Suicide Scale^22^ focused on planning and preparatory behavior (ie, considered a specific suicide method, identified how to acquire your suicide method, made other preparation for your death [eg, wrote a suicide note, made arrangements]). These items were also rated on a 5-point Likert scale (from 1 [not at all] to 5 [very much]). Each morning, participants rated subjective sleep quality on a 5-point Likert scale (from 1 [very bad night of sleep] to 5 [very good night of sleep]).

### Nighttime Phone Use

We operationalized nighttime phone use in 3 distinct ways, progressing from coarse to fine-grained, given previous work suggesting that different nighttime phases may uniquely impact sleep and psychological functioning^9,14^: (1) maximum phone-free gap (8:00 pm to 10:00 am), (2) use during self-reported sleep windows, and (3) hourly use bins (11:00 pm to 8:00 am). For study 1, we first calculated the longest phone-free interval, termed the maximum nighttime phone-free gap and operationalized as the largest gap between screenshots within a fixed 8:00 pm to 10:00 am window for each night. If no screenshots were detected, the gap was set to the full 14-hour window. This daily gap value was linked to all completed EMA entries provided on that day. Screenshots for the 24 hours preceding each participant’s first EMA of the day were extracted. The eMethods in Supplement 1 provide the full data processing details.

For study 2, nighttime use was defined based on each participant’s self-reported sleep interval. Sleep entries were manually cleaned and preprocessed using a Python, version 3.9.6 (Python Software Foundation) pipeline that standardized time formats, filtered invalid responses, and adjusted for windows crossing midnight. Only EMA responses with corresponding sleep data were included in this analysis. Two primary nighttime use covariates were computed: phone minutes and keyboard minutes during the sleep window. To control for overall usage, phone minutes and keyboard minutes were calculated for the preceding 24-hour period, excluding the sleep window. The eMethods in Supplement 1 provide the full data processing details, and eFigure 1 in Supplement 1 details the association between self-reported sleep hours and the maximum nighttime phone-free gap.

For study 3, to examine potential time-dependent associations of nighttime smartphone use, EMA responses were linked to phone use data from specific nighttime hours (11:00 pm to 8:00 am), defined based on typical sleep patterns in the sample (see study 2). Previous-day daytime usage (8:00 am to 11:00 pm) was calculated as a control and included for both phone and keyboard activity.

To test whether the association between smartphone use and suicidal ideation or suicide planning varied by time of night, the variable hour of night was grouped into 3 bins: late (11:00 pm to 1:00 am), middle (1:00 to 5:00 am), and early (5:00 to 8:00 am). We also specified random effects for each hour of the night, which did not improve model fit (mean [SE] change in leave-one-out cross-validation information criterion, −1.1 [3.2] for passive suicidal ideation, −0.4 [4.0] for active suicidal ideation, and −1.8 [2.6] for suicide planning), but for descriptive purposes, refer to eFigures 2 to 4 in Supplement 1.

### Keyboard Presence

We used keyboard presence as a proxy for active use. To create this variable, we first manually annotated a random sample of 1000 screenshots for keyboard presence (490 positive cases) to create a labeled training set. Using this dataset, we developed a deep learning classifier based on the EfficientNet-B2 architecture,^23^ using data augmentation and early stopping to improve generalizability. The model was trained on 70% of the data, validated on 15%, and achieved perfect classification accuracy on a 150-image test set. Applied to the full dataset of 7.5 million screenshots, the model detected keyboard presence in 1.05 million images (14%). Each screenshot in the raw dataset (prior to study-specific preprocessing) was coded for keyboard presence as a binary variable (0 = absent, 1 = present).

### Statistical Analysis

Analyses were conducted using the brms package^24^ in R, version 4.4.1 (R Foundation for Statistical Computing). Multilevel models were used to account for the nonindependence of observations. For passive suicidal ideation and active suicidal ideation, cumulative logistic multilevel models (ordinal models) were specified. Suicide planning was dichotomized (presence vs absence of planning) due to approximately 13% nonzero responses, and a Bernoulli logistic multilevel model was used. All reported bayesian models were run using 4 Markov chains, with at least 1000 post-warmup samples saved per chain. Model convergence was assessed using R-hat values. Default priors specified by brms were used unless otherwise noted. Instead of *P* values, statistical significance was determined based on whether the 95% credible interval (CrI) for a parameter estimate excluded 0.

Time-varying covariates (nighttime phone or keyboard use metrics, previous day phone or keyboard use metrics, and subjective sleep scores) were calculated using *z* scores and decomposed into between-person average and within-person average, which were included simultaneously as fixed effects. All models also included random intercepts for participants to account for baseline individual differences. For the study 1 analysis, previous-day phone use (total number of screenshots during the preceding calendar day) was also included in the model.

All multilevel models used listwise deletion for observations with missing values on any variable included in the specific model. To link nighttime variables to EMA scores that varied across the next day, each nighttime score was linked to each complete EMA response from the next day (as opposed to just the first response of the day). The eMethods in Supplement 1 provide additional analysis details.

## Results

The sample included 79 participants (mean [SD] age, 35.2 [11.1] years; 54 female [68.3%] and 25 male [31.7%]; 5 self-identifying as American Indian or Alaska Native [6.3%], 5 as Black or African American [6.3%], 67 as White [84.8%], 1 as multiracial [1.3%], and 1 not listed [1.3%] race; 6 self-identifying as Hispanic/Latino [7.6%] and 73 as non-Hispanic/Latino ethnicity [92.4%]) (Table). With regard to clinical presentation, 67 participants (91.8%) reported a lifetime history of individual psychotherapy, and 65 (89.0%) reported a lifetime history of having taken prescription medication for mental health or emotional reasons. The overall EMA compliance rate was 68.8%. Nonzero passive suicidal ideation was reported on 37.1% of nonmissing observations (1653 of 4458; mean [SD], 1.04 [1.71]; range, 0-8), nonzero active suicidal ideation on 35.0% of nonmissing observations (1560 of 4458; mean [SD], 0.84 [1.48]; range, 0-8), and nonzero suicide planning on 13.0% of nonmissing observations (580 of 4458; mean [SD], 0.33 [1.19]; range, 0-12).

**Table.** Full-Sample Characteristics (N = 79)

Characteristic	No. (%)
Age, mean (SD), y	35.2 (11.1)
Sex	
Female	54 (68.3)
Male	25 (31.7)
Race	
American Indian or Alaska Native	5 (6.3)
Black or African American	5 (6.3)
White	67 (84.8)
Multiracial	1 (1.3)
Not listed^a^	1 (1.3)
Ethnicity	
Hispanic/Latino	6 (7.6)
Non-Hispanic/Latino	73 (92.4)
Employment status	
Full-time	35 (44.3)
Part-time	9 (11.4)
Student	7 (8.9)
Unemployed	28 (35.4)
Income, $	
≤19 000	19 (24.1)
20 000-39 000	26 (32.8)
40 000-59 000	19 (24.1)
60 000-79 000	10 (12.7)
≥80 000	5 (6.3)
Highest level of education	
High school or less	15 (18.9)
Some college	22 (27.9)
Technical or business school	4 (5.1)
College graduate	25 (31.6)
Some graduate school or advanced degree	13 (16.5)
Current diagnostic presentation	
Major depressive disorder	34 (43.0)
Generalized anxiety disorder	27 (34.2)
Panic disorder	32 (40.5)
Social anxiety disorder	20 (25.3)
Posttraumatic stress disorder	26 (32.9)
Alcohol use disorder	18 (22.8)
Substance use disorder	20 (25.3)
Suicide risk history	
Past-week suicidal ideation	31 (39.2)
Lifetime suicide plan	57 (72.2)
Past-year suicide plan	30 (38)
Lifetime suicide attempt	51 (64.5)
Past-year suicide attempt	10 (12.7)

^a^ Participant did not list an alternative race.

### Temporal Patterns of Nighttime Smartphone Use

**Figure 1.**
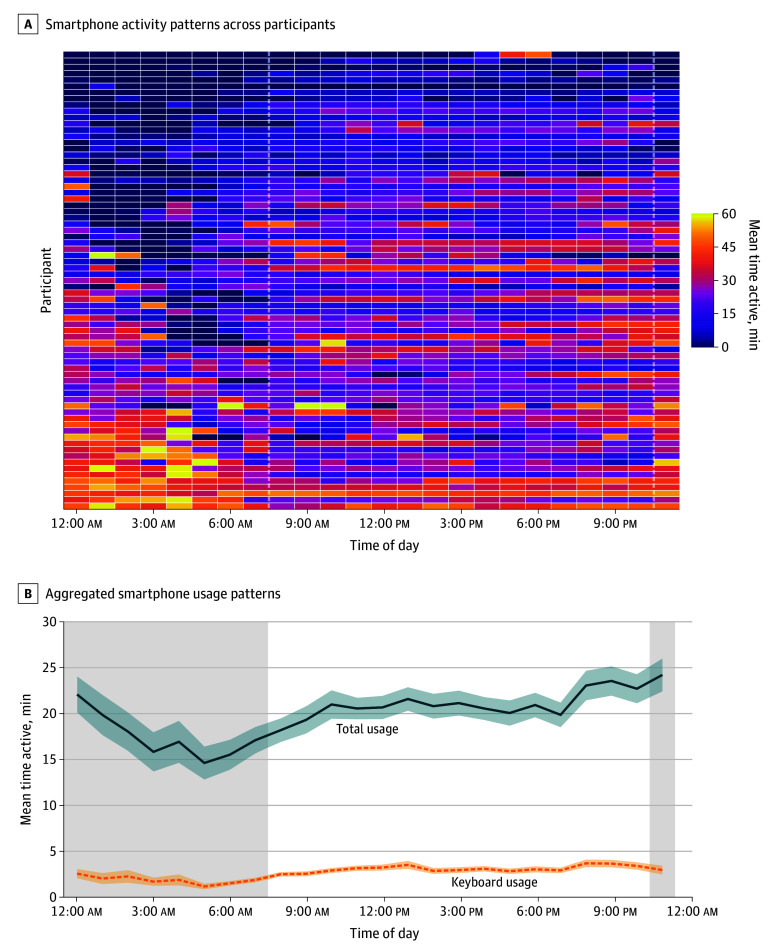
Smartphone Activity Patterns Across a 24-Hour Period A, Each row of the heat map represents 1 participant, and the color intensity indicates usage level (0 to ≥60 minutes per hour). B, Shaded regions indicate nighttime hours (11:00 pm to 8:00 am).

Figure 1 illustrates 24-hour smartphone activity patterns across all participants. Participants (774 observations) reported moderate levels of subjective sleep quality (mean [SD], 2.7 [1.1]). For this variable, we only included a between-person representation given the response rate (37.9%). Substantial individual variation in nighttime phone use was observed, with distinct behavioral phenotypes: concentrated late-night users (11:00 pm to 1:00 am), distributed nocturnal users with scattered activity throughout the night, and minimal nighttime users (Figure 1A). Aggregated patterns showed phone use declining after 11:00 pm, reaching its nadir at approximately 4:00 am before increasing in the early-morning hours (Figure 1B). Keyboard usage followed a similar, but attenuated pattern, indicating reduced active engagement during nighttime hours.

### Study 1: Maximum Nighttime Phone-Free Gap

**Figure 2.**
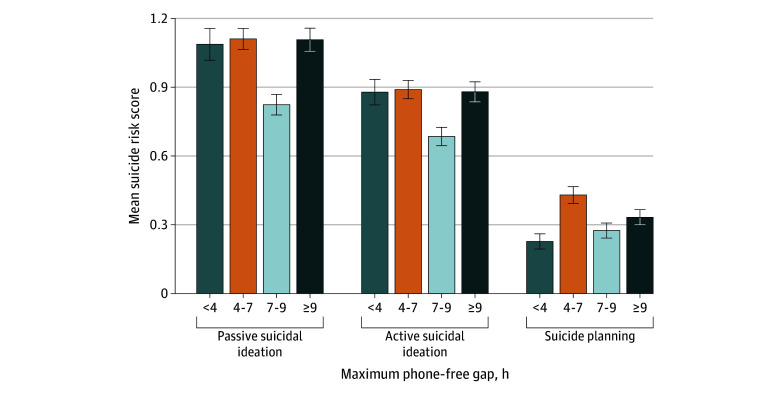
Maximum Nighttime Phone-Free Gap and Next-Day Suicide Risk Indicators Scored on a 5-point Likert scale of 1 (not at all) to 5 (very much). Error bars indicate SEs.

Across 70 participants, 1207 maximum nighttime phone-free gap values were calculated (calculation required ≥1 EMA response on the next day; mean [SD], 16.5 [8.8] responses per participant), and when linked to EMA responses, yielded 4439 rows. The mean (SD) maximum gap was 7.66 (3.52) hours (median [IQR], 7.30 [5.09-9.87] hours) (eFigure 5 in Supplement 1). To study the potential nonlinear association with gap duration, we categorized maximum gap duration into 4 ranges (<4 hours, 4-7 hours, 7-9 hours, ≥9 hours), with the following distribution: <4 hours, 608 rows; 4 to 7 hours, 1452 rows; 7 to 9 hours, 1038 rows; 9 or more hours, 1338 rows. We also fit models with a spline for gap duration, although these did not improve fit (mean [SE] change in leave-one-out cross-validation information criterion, −5.7 [4.2] for passive suicidal ideation, −5.6 [4.2] for active suicidal ideation, and −1.3 [2.0] for suicide planning). For descriptive purposes, mean next-day suicide risk indicators across these groups are displayed in Figure 2.

Higher between-person subjective sleep quality was significantly associated with lower passive suicidal ideation and active suicidal ideation. For passive suicidal ideation, compared with the reference category of 7- to 9-hour gaps, participants with 4- to 7-hour gaps showed significantly higher levels of passive suicidal ideation (B = 0.35; 95% CrI, 0.14-0.55), while gaps of less than 4 hours and at least 9 hours showed no significant differences in passive suicidal ideation from the 7- to 9-hour reference category. For active suicidal ideation, participants with 4- to 7-hour gaps also showed significantly higher levels of active suicidal ideation compared with the 7- to 9-hour reference (B = 0.29; 95% CrI, 0.10-0.49), while other gap categories showed no significant differences. No associations were found for previous-day row count variables for either passive or active suicidal ideation (eTables 1 and 2 in Supplement 1). For suicide planning, only between-person subjective sleep quality had a significant association (B = −0.57; 95% CrI, −1.13 to −0.03) (eTable 3 in Supplement 1).

### Study 2: Self-Reported Sleep Windows

Seventy-five participants (96.2%) provided at least 2 valid entries, with a mean [SD] of 14.3 [7.3] reports per participant, yielding 3012 rows for data analysis. Results indicated that higher between-person subjective sleep quality was significantly associated with lower passive suicidal ideation (B = −0.96; 95% CrI, −1.57 to −0.34) and lower active suicidal ideation (B = −1.02; 95% CrI, −1.59 to −0.44). For passive suicidal ideation, higher within-person sleep phone minutes were significantly associated with higher levels of passive suicidal ideation (B = 0.15; 95% CrI, 0.04-0.26), while higher within-person sleep keyboard minutes (B = −0.14; 95% CrI, −0.24 to −0.04) and higher within-person nonsleep keyboard minutes (B = −0.16; 95% CrI, −0.29 to −0.03) were significantly associated with lower levels of passive suicidal ideation. For active suicidal ideation, higher within-person sleep keyboard minutes were significantly associated with lower levels of active suicidal ideation (B = −0.13; 95% CrI, −0.24 to −0.02), while higher within-person nonsleep keyboard minutes showed a significant association with lower levels active suicidal ideation (B = −0.12; 95% CrI = −0.24 to −0.001). No associations were found for any between-person components of phone or keyboard minutes during or outside of self-reported sleep windows for either passive suicidal ideation (eTable 4 in Supplement 1) or active suicidal ideation (eTable 5 in Supplement 1). For suicide planning, no associations were found outside a negative association with between-person subjective sleep quality (B = −0.73; 95% CrI, −1.42 to −0.05) (eTable 6 in Supplement 1).

### Study 3: Hourly Nighttime Use

**Figure 3.**
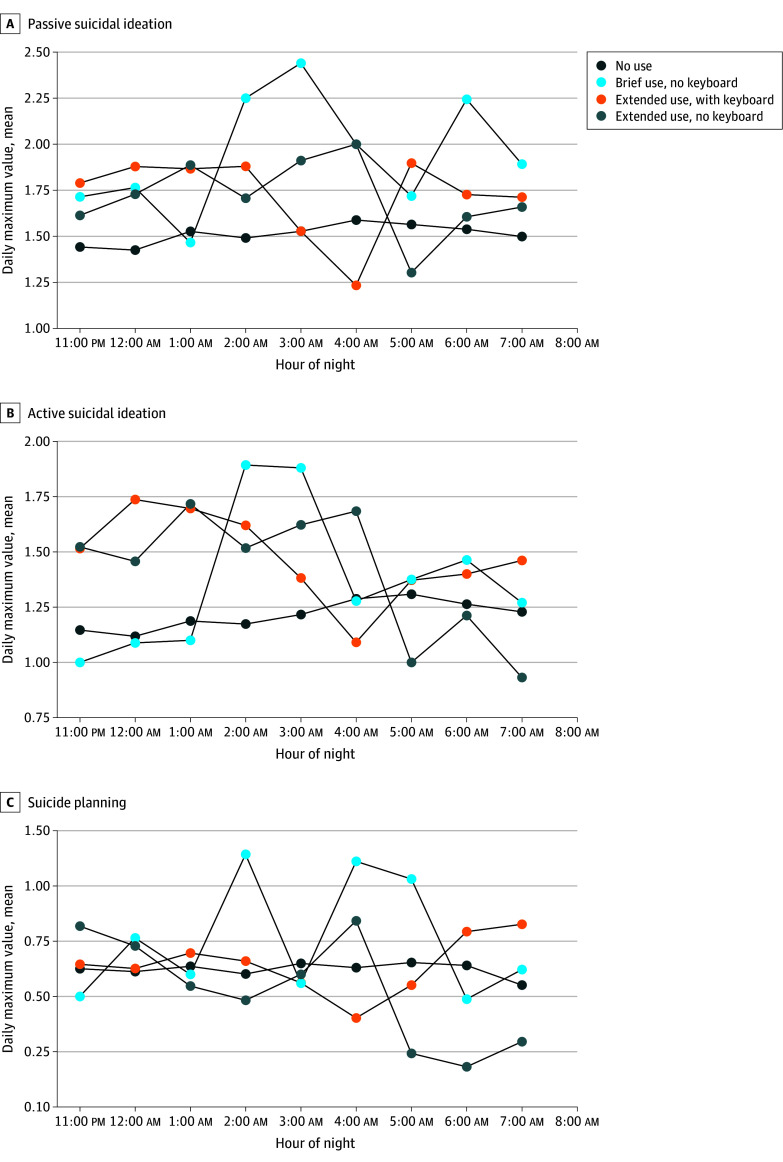
Hourly Nighttime Smartphone Use and Next-Day Suicide Risk Indicators Hourly nighttime phone use was categorized based on passively collected screenshots as follows: (1) no phone use (no screenshots detected), (2) brief use (<5 minutes of total screen time), and (3) extended use (≥5 minutes). Keyboard presence was used to further differentiate active phone engagement within usage categories. For each day, the maximum score was taken for passive suicidal ideation, active suicidal ideation, and suicide planning; from these, the mean of the maximum scores across days for the corresponding variable was calculated.

The resultant data set had 29 781 rows across 67 participants and 3308 EMA responses, with the following number of unique phone use values in each hourly bin: 6618 for late (11:00 pm to 1:00 am), 13 236 for middle (1:00 am to 5:00 am), and 9927 for early (5:00 am to 8:00 am) (full data processing details in the eMethods in Supplement 1). To visualize patterns of hourly nighttime phone use compared with suicide risk indicators, we categorized each sleep period based on the presence and duration of detected screen activity, including no use, brief use (<5 minutes), or extended use (≥5 minutes), and further distinguished periods with keyboard input. Mean levels of next-day passive suicidal ideation, active suicidal ideation, and suicide planning are displayed across these categories in Figure 3.

Higher within-person hourly nighttime phone use (B = 0.13; 95% CrI, 0.08-0.18) and previous-day phone use (B = 0.08; 95% CrI, 0.04-0.12) was significantly associated with higher levels of passive suicidal ideation. Higher within-person previous-day keyboard use (B = −0.23; 95% CrI, −0.27 to −0.19) and higher between-person subjective sleep quality (B = −1.03; 95% CrI, −1.65 to −0.40) were significantly associated with lower levels of passive suicidal ideation. The association between within-person phone use and passive suicidal ideation had significant interactions with time bins, in which it was significantly more negative (eg, less risk) during the middle-night bin (simple slope, 0.04; 95% CrI, −0.01 to 0.07) and early-morning bin (simple slope, −0.07; 95% CrI, −0.12 to −0.01) compared with the late-night bin. Additionally, within-person keyboard use showed a significant interaction, with its association with passive suicidal ideation being significantly more negative (eg, less risk) during the middle-night bin compared with the late-night reference bin (simple slope, −0.10; 95% CrI, −0.13 to −0.07). No other main effects were significant (eTable 7 and eFigures 6 and 7 in Supplement 1).

For active suicidal ideation, higher between-person hourly nighttime phone use (B = 2.27; 95% CrI, 0.03-4.52), higher within-person hourly nighttime phone use (B = 0.10; 95% CrI, 0.05-0.15), higher between-person previous-day keyboard use (B = 1.57; 95% CrI, 0.14-2.98), and within-person previous-day phone use (B = 0.08; 95% CrI, 0.04-0.12) were significantly associated with higher levels of active suicidal ideation. Conversely, higher between-person previous-day phone use (B = −1.73; 95% CrI, −3.18 to −0.38), higher within-person previous-day keyboard use (B = −0.23; 95% CrI, −0.27 to −0.19), and higher between-person subjective sleep (B = −1.08; 95% CrI, −1.67 to −0.54) were significantly associated with lower levels of active suicidal ideation. A significant interaction was found for within-person nighttime hourly phone use, in which compared with the late-night bin, the association between higher within-person phone use and levels of active suicidal ideation was significantly more negative (eg, less risk) during the middle-night bin (simple slope, −0.04; 95% CrI, −0.08 to 0.001) and early-morning bin (simple slope, −0.12; 95% CrI, −0.18 to −0.07) (eFigure 8 in Supplement 1). No other interactions were significant (eTable 8 in Supplement 1).

**Figure 4.**
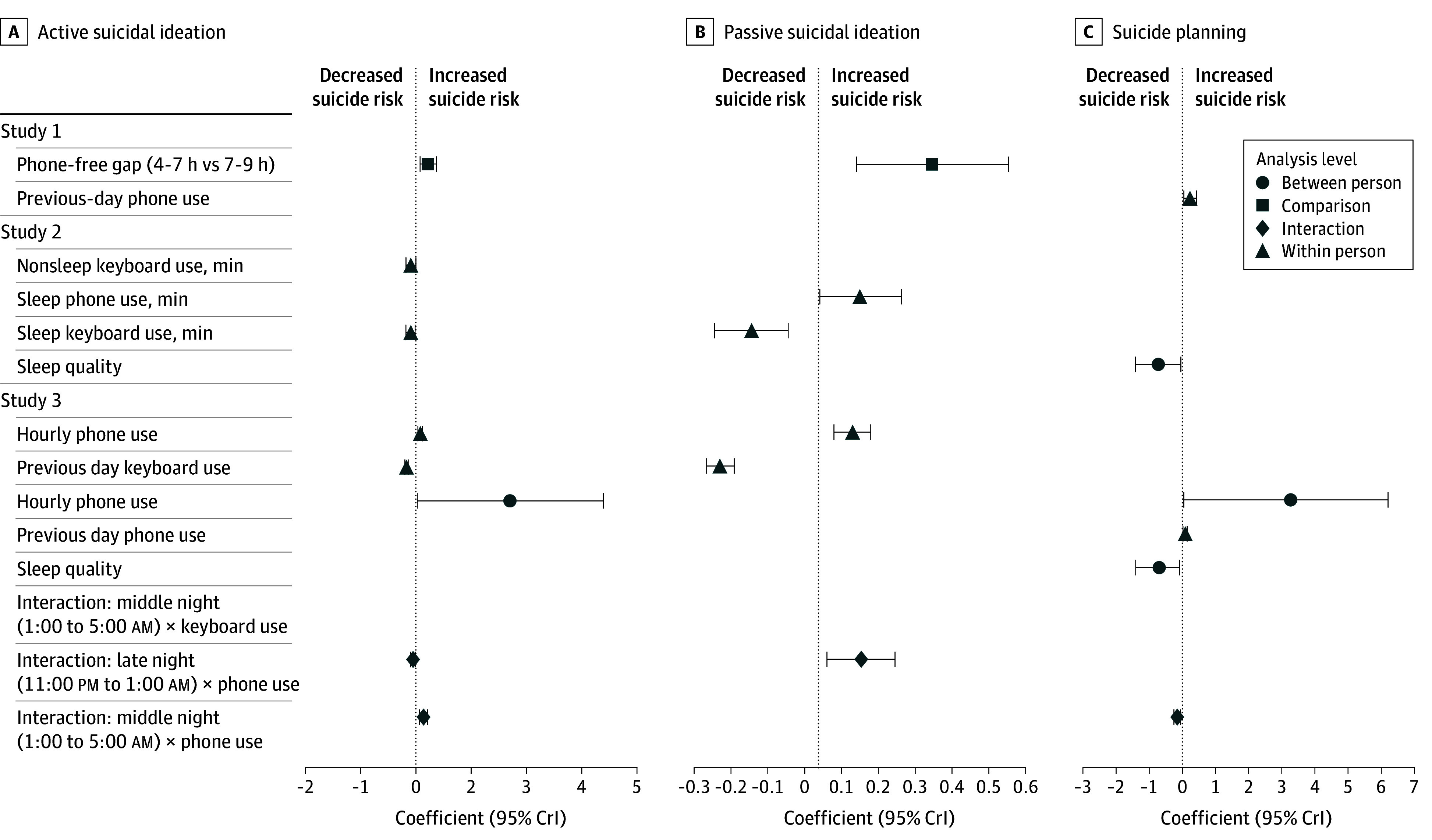
Associations Between Nighttime Phone-Use Metrics and Next-Day Suicide Risk Indicators The forest plot displays estimated regression coefficients and 95% credible intervals (CrIs) from mixed-effects models estimating the 3 suicide outcomes.

For suicide planning, higher within-person previous-day phone use (B = 0.08; 95% CrI, 0.02-0.15) was significantly associated with higher odds of suicide planning, while higher within-person previous-day keyboard use (B = −0.06; 95% CrI, −0.13 to −0.002) and higher between-person subjective sleep (B = −0.70; 95% CrI, −1.41 to −0.09) were significantly associated with lower odds of suicide planning. Higher between-person hourly nighttime phone use was significantly associated with higher odds of suicide planning (B = 3.26; 95% CrI, 0.04-6.21). The association between within-person nighttime phone use and odds of suicide planning had significant interactions with time bins, in which it was significantly more negative (eg, less risk) in both the middle-night bin (simple slope, −0.08; 95% CrI, −0.14 to −0.02) and the early-morning bin (simple slope, −0.10; 95% CrI, −0.18 to −0.02) compared with the late-night bin. No other main effects or interactions were significant (eTable 9 and eFigure 9 in Supplement 1). A summary of all findings across all studies is presented in Figure 4.

## Discussion

This study combining surveys and passive data collection found that the timing and type of nighttime smartphone use are meaningfully associated with next-day suicidal ideation and suicide planning. With the use of a high-frequency digital phenotyping approach and EMA across 28 days, we identified temporally and behaviorally specific markers of suicidal ideation and suicide planning. Our analyses showed that using coarse indicators alone (eg, total phone use) did not fully capture these dynamics, as what mattered was when and how smartphones are used during the night.

The study consistently found that late-night phone use (11:00 pm to 1:00 am) was associated with higher next-day suicide risk. In study 3, greater phone use during this window, particularly compared with middle-night (1:00 to 5:00 am) or early-morning (5:00 to 8:00 am) use, was associated with significantly elevated passive and active suicidal ideation and suicide planning. These findings align with growing concerns that the late-night period (eg, the hours prior to bedtime) represents a critical risk window, potentially due to reduced social support, increased cognitive rumination, or exposure to distressing content.^9,14^ Phone use during these hours may also impact sleep onset or quality due to blue light exposure.^6,7^ Future work needs to replicate these findings and could benefit from further exploration of the potentially protective effects of middle-night active engagement, including which applications and application content may be influential.

Complementing these findings, study 1 showed that the duration of nighttime phone inactivity, a potential proxy for uninterrupted rest, was also associated with suicidal ideation. Specifically, 7- to 9-hour phone-free intervals were associated with the lowest levels of next-day ideation, while gaps of 4 to 7 hours were associated with a significantly elevated risk for suicidal ideation. These results identified 4- to 7-hour phone-free intervals as a specific risk window, with only this range showing significantly elevated suicidal ideation compared with the 7- to 9-hour reference group, while both shorter (<4 hours) and longer (≥9 hours) gaps showed no significant differences. These findings suggest that disrupted, but not entirely absent sleep may be particularly problematic but need replication in a larger sample.

Descriptive data (Figure 1) further revealed distinct nighttime usage phenotypes across participants, including concentrated late-night usage, diffuse nocturnal usage, and minimal nighttime usage. These preliminary phenotypes highlight the potential heterogeneity in nighttime behaviors, reinforcing the value of high-resolution data in identifying personalized risk patterns.

Beyond timing, our findings provide initial support for the importance of engagement type. In both studies 2 and 3, keyboard presence, as a proxy for active use (eg, messaging, typing), was associated with lower suicidal ideation, particularly during the middle of the night. Keyboard activity during self-reported sleep windows was significantly associated with lower passive and active suicidal ideation at the within-person level. In study 3, significant time-by-use interactions showed that the positive association between phone use and suicide risk during late-night hours (11:00 pm to 1:00 am) was significantly attenuated or reversed during the middle-night (1:00 to 5:00 am) and early-morning periods.

These findings are congruent with growing evidence that active engagement may serve a regulatory or social function, offering distraction, connection, or emotional processing during periods of distress.^15,16^ In contrast, passive use, such as prolonged scrolling, may increase the risk of suicidal ideation through mechanisms such as social comparison, emotional disengagement, or disrupted sleep.^14^ The nuanced behavioral profiles identified here offer an early indication that not all phone use is harmful; some patterns may actually serve as coping strategies.

### Limitations

Several limitations merit consideration. First, our study lacked objective sleep tracking, so phone inactivity may reflect rest or disengagement, not necessarily sleep, and limited our ability to examine more personalized models. Second, keyboard presence, while a scalable and interpretable proxy, does not capture the content or valence of use, which limited our ability to distinguish between helpful and harmful forms of engagement. Third, the observational nature of our study precluded causal inference; suicidal thoughts may drive late-night phone use, not just result from it. Fourth, while we modeled both within-person and between-person effects, our focus on next-day associations may have missed risk processes that unfold more rapidly or accumulate over time. Fifth, the high degrees of missingness for self-reported bedtimes and wake times (averaging 14.3 reports per participant) and subjective sleep quality (37.9% response rate) may not be missing at random, as participants experiencing more severe sleep disruption or heightened suicide risk may have been less likely to complete these assessments, potentially biasing our estimates of sleep-suicide associations. Finally, our sample was limited to Android smartphone users with recent suicidal thoughts, constraining the generalizability of the specific associations. Furthermore, to have implications for prevention efforts, findings will need to be replicated in a more general sample, including individuals without recent suicidal thoughts.

## Conclusions

This study combining surveys and passive data collection suggests that among individuals with recent suicidal thoughts, nighttime phone use may not be uniformly risky; rather, suicidal ideation and suicide planning may depend on when and how individuals engage with their devices. As such, interventions that seek to simply reduce overall use may overlook meaningful distinctions between harmful and potentially adaptive behaviors. Upon replication of these findings, behavioral signals, such as late-night passive use, could be incorporated into real-time risk detection algorithms or used to trigger just-in-time adaptive interventions. As the field advances toward scalable, personalized mental health tools, integrating fine-grained passive data with dynamic self-report holds promise for improving suicide prevention efforts that target suicidal ideation and suicide planning.

## Notes

Supplement 1.
eMethods.
**eTable 1. **Study 1 Variables Estimating Passive Suicidal Ideation (Categorical Gap Model) **eTable 2. **Study 1 Variables Estimating Active Suicidal Ideation (Categorical Gap Model) **eTable 3. **Study 1 Variables Estimating Suicide Planning (Categorical Gap Model) **eTable 4.** Study 2 Variables Estimating Passive Suicidal Ideation **eTable 5.** Study 2 Variables Estimating Active Suicidal Ideation **eTable 6.** Study 2 Variables Estimating Suicide Planning (Binary) **eTable 7.** Study 3 Variables Estimating Passive Suicidal Ideation **eTable 8.** Study 3 Variables Estimating Active Suicidal Ideation **eTable 9.** Study 3 Variables Estimating Suicide Planning (Binary) **eFigure 1.** Association Between Maximum Phone-Free Gap and Self-Reported Sleep Duration **eFigure 2.** Random Effects of Hour of Night in Estimating Passive Suicidal Ideation **eFigure 3.** Random Effects of Hour of Night in Estimating Active Suicidal Ideation **eFigure 4.** Random Effects of Hour of Night in Estimating Passive Suicide Planning **eFigure 5.** Distribution of Maximum Nighttime Phone-Free Gap **eFigure 6.** Interaction for Within-Person Hourly Nighttime Smartphone Use Estimating Passive Suicidal Ideation **eFigure 7.** Interaction for Within-Person Hourly Nighttime Keyboard Use Estimating Passive Suicidal Ideation **eFigure 8.** Interaction for Within-Person Hourly Nighttime Smartphone Use Estimating Active Suicidal Ideation **eFigure 9.** Interaction for Within-Person Hourly Nighttime Smartphone Use Estimating Suicide Planning

Supplement 2.
Data Sharing Statement
