# Supplementary material for: Passive vs Active Nighttime Smartphone Use as Markers of Next-Day Suicide Risk
Source: JAMA Netw Open. 2025 Nov 11;8(11):e2542675. doi: 10.1001/jamanetworkopen.2025.42675 (PMC12606377; doi:10.1001/jamanetworkopen.2025.42675)
Supplement: Supplement 1. — eMethods. eTable 1. Study 1 Variables Estimating Passive Suicidal Ideation (Categorical Gap Model) eTable 2. Study 1 Variables Estimating Active Suicidal Ideation (Categorical Gap Model) eTable 3. Study 1 Variables Estimating Suicide Planning (Categorical Gap Model) eTable 4. Study 2 Variables Estimating Passive Suicidal Ideation eTable 5. Study 2 Variables Estimating Active Suicidal Ideation eTable 6. Study 2 Variables Estimating Suicide Planning (Binary) eTable 7. Study 3 Variables Estimating Passive Suicidal Ideation eTable 8. Study 3 Variables Estimating Active Suicidal Ideation eTable 9. Study 3 Variables Estimating Suicide Planning (Binary) eFigure 1. Association Between Maximum Phone-Free Gap and Self-Reported Sleep Duration eFigure 2. Random Effects of Hour of Night in Estimating Passive Suicidal Ideation eFigure 3. Random Effects of Hour of Night in Estimating Active Suicidal Ideation eFigure 4. Random Effects of Hour of Night in Estimating Passive Suicide Planning eFigure 5. Distribution of Maximum Nighttime Phone-Free Gap eFigure 6. Interaction for Within-Person Hourly Nighttime Smartphone Use Estimating Passive Suicidal Ideation eFigure 7. Interaction for Within-Person Hourly Nighttime Keyboard Use Estimating Passive Suicidal Ideation eFigure 8. Interaction for Within-Person Hourly Nighttime Smartphone Use Estimating Active Suicidal Ideation eFigure 9. Interaction for Within-Person Hourly Nighttime Smartphone Use Estimating Suicide Planning [file jamanetwopen-e2542675-s001.pdf]

Supplemental Online Content

Jacobucci R, Jones S, Blacutt M, Ammerman BA. Passive vs active nighttime smartphone use as markers of next-day suicide risk. *JAMA Netw Open*. 2025;8(11):e2542675. doi:10.1001/jamanetworkopen.2025.42675

eMethods
eTable 1. Study 1 Variables Estimating Passive Suicidal Ideation (Categorical Gap Model)
eTable 2. Study 1 Variables Estimating Active Suicidal Ideation (Categorical Gap Model)
eTable 3. Study 1 Variables Estimating Suicide Planning (Categorical Gap Model)
eTable 4. Study 2 Variables Estimating Passive Suicidal Ideation
eTable 5. Study 2 Variables Estimating Active Suicidal Ideation
eTable 6. Study 2 Variables Estimating Suicide Planning (Binary)
eTable 7. Study 3 Variables Estimating Passive Suicidal Ideation
eTable 8. Study 3 Variables Estimating Active Suicidal Ideation
eTable 9. Study 3 Variables Estimating Suicide Planning (Binary)
eFigure 1. Association Between Maximum Phone-Free Gap and Self-Reported Sleep Duration
eFigure 2. Random Effects of Hour of Night in Estimating Passive Suicidal Ideation
eFigure 3. Random Effects of Hour of Night in Estimating Active Suicidal Ideation
eFigure 4. Random Effects of Hour of Night in Estimating Passive Suicide Planning
eFigure 5. Distribution of Maximum Nighttime Phone-Free Gap
eFigure 6. Interaction for Within-Person Hourly Nighttime Smartphone Use Estimating Passive Suicidal Ideation
eFigure 7. Interaction for Within-Person Hourly Nighttime Keyboard Use Estimating Passive Suicidal Ideation
eFigure 8. Interaction for Within-Person Hourly Nighttime Smartphone Use Estimating Active Suicidal Ideation
eFigure 9. Interaction for Within-Person Hourly Nighttime Smartphone Use Estimating Suicide Planning

This supplemental material has been provided by the authors to give readers additional information about their work.

© 2025 Jacobucci R et al. *JAMA Network Open*.

eMethods

Reporting and Transparency

This report follows the American Association for Public Opinion Research (AAPOR) reporting guideline for survey studies, including the Transparency Initiative and *Standard Definitions* (10th ed.). We disclose the study sponsor/funder (R21MH129688), the organizations and investigators that designed/ran the study, target population and eligibility, recruitment sources and procedures (nonprobability convenience sample), field dates (August 2022–January 2024), modes/language (smartphone EMA in English; passive screenshot capture), instruments with exact item wording, incentives, data-quality procedures, sample size/compliance, and weighting (none). We report AAPOR-style final disposition codes and study-level outcome rates (see Table), including completes (I), partials (P), other eligible non-interviews (O), ineligible (IE), and unknown eligibility (UO), with cooperation (I/[I+P+R+O]) calculated per *Standard Definitions* (10th ed.). Because this is a nonprobability sample intended for etiologic modeling rather than population estimation, we do not report a margin of sampling error; statistical uncertainty is conveyed via Bayesian 95% credible intervals.

Table. AAPOR-Style Final Dispositions and Outcome Rates for Study Recruitment

AAPOR Code	**Disposition Category**	**n**	**% of total**
**I**	Complete interview (completed baseline and began EMA; included in analyses)	79	11.0
**P**	Partial/break-off after start (withdrew early)	4	0.6
**R**	Refusal/break-off among eligibles	0	0.0
**O**	Other eligible, not interviewed (eligible at screen; baseline no-show/reschedule)	50	7.0
**IE**	Ineligible after screening	251	35.0
**UO**	Unknown eligibility (expressed interest/no follow-up; scheduled screen not completed)	334	46.5
Total	**All initial cases**	**718**	**100.0**

© 2025 Jacobucci R et al. *JAMA Network Open*.

General Method: Data Analysis

Model Selection and Rationale. We initially evaluated zero-inflated Poisson (ZIP) mixed-effects models to address the count nature and high proportion of zero responses in suicidal ideation and suicidal planning variables. However, these models demonstrated convergence issues and unstable parameter estimates across multiple studies. We also explored frequentist ordinal models (e.g., using the ordinal package in R) for the suicidal ideation outcomes but similarly encountered convergence difficulties.

As a result, we selected Bayesian multilevel models implemented via the brms package (Bürkner, 2017), which offered greater flexibility, improved model stability, and interpretability for our research questions.

- For passive and active suicidal ideation, we used cumulative logistic (ordinal) models, treating the Likert-style scales as ordered categorical variables.
- For suicidal planning, due to low non-zero response rates (~10% across studies), we dichotomized the variable (presence vs. absence of planning) and used a Bernoulli logistic model to estimate the probability of planning occurrence.

Model Estimation and Diagnostics. All Bayesian models were estimated in R (R Core Team, 2024) using 4 Markov chains. Each chain saved at least 1,000 post-warmup samples (e.g., 1,500 total iterations with 750 warmup, or 2,000 with 1,000 warmup, depending on model complexity). Convergence was assessed via R-hat values, with values <1.01 indicating acceptable convergence. Default weakly informative priors from brms were used unless otherwise specified. Rather than p-values, parameter significance was assessed via 95% Credible Intervals (CIs); a parameter was considered meaningful if its CI excluded zero.

© 2025 Jacobucci R et al. *JAMA Network Open*.

Multilevel Structure and Variable Decomposition. All models included random intercepts for participants to account for individual baseline differences. To distinguish between stable between-person (BP) effects and dynamic within-person (WP) fluctuations, we decomposed key time-varying covariates—including nighttime phone/keyboard use, prior day phone use, and subjective sleep quality—into separate BP and WP components. Both were included as fixed effects in the multilevel models.

Missing Data. Item-level missingness within completed EMA surveys was negligible (<1%), so no imputation was used for individual survey items. Prompt-level missingness was handled inherently by the multilevel modeling framework, which uses all available observations per participant. No imputation was applied to EMA outcome variables or subjective sleep scores, as the focus was not on reconstructing entire behavioral sequences or missing study days.

Study 1: Method

To operationalize nighttime smartphone inactivity as a proxy for sleep continuity, we calculated the **maximum phone-free gap** using passively collected screenshot data. The nighttime window was defined as 8:00 PM to 10:00 AM based on self-reported sleep data from Study 1. For each calendar day, we first identified the timestamp of the participant's **earliest EMA response,** and then extracted all screenshots that fell within the **preceding 24-hour nighttime window**.

Within this filtered set of nighttime screenshots, **timestamps were sorted chronologically,** and the time difference between each pair of consecutive screenshots was calculated in seconds. The **primary covariate,** *maximum nighttime phone-free gap,* was defined as the longest of these intervals, converted to hours. If **fewer than two screenshots** were recorded in the defined window, or if the maximum gap could not be calculated due to data

sparsity, a default value of **14 hours** was assigned. Additionally, any calculated gaps exceeding 14 hours were also **capped at 14 hours** to reflect the bounds of the defined nighttime window. See eFigure 1 for distribution of maximum nighttime phone-free gap.

To study the potential nonlinear association of gap duration, we categorized maximum gap duration into four ranges (<4 hrs, 4–7 hrs, 7–9 hrs, ≥9 hrs), with the following distribution: (<4 hrs = 608; 4–7 hrs = 1,452; 7–9 hrs = 1,038; ≥9 hrs = 1,338). These categories were determined based on a combination of the data distribution and the Centers for Disease Control and Prevention's recommendations for adults, which include 7 or more hours of sleep and 7-9 hours of sleep for specific age groups.

To control for general phone usage patterns, we computed a covariate—**prior day phone use**—as the total number of screenshots recorded during the **calendar day immediately preceding** the EMA date. Both the maximum phone-free gap and prior day phone use variables were **standardized (z-scored)** and **decomposed into between-person and within-person components** for use in multilevel modeling.

Study 2: Method

Sleep Window Preprocessing. Self-reported sleep window data were manually reviewed and standardized using a Python-based preprocessing pipeline. This pipeline was designed to robustly handle a variety of time formats (e.g., 12-hour, 24-hour, with or without AM/PM designations) and applied regular expression pattern matching to filter invalid entries. Entries with missing or nonsensical bedtime or wake time values were removed. Valid time entries were converted to minutes since midnight and standardized across participants. For sleep windows that crossed midnight, date adjustments were applied to ensure continuity of the nighttime period. Following preprocessing, 1,114 valid sleep window observations were retained from 78

participants. Of these, 75 participants (96.2%) provided at least two valid entries, with an average of 14.3 valid reports per participant.

Descriptive Sleep Characteristics. Substantial variability was observed in sleep schedules. On weekdays (n = 834 nighttime observations), the average reported sleep duration was 8.92 hours (SD = 4.35 hours). The circularly averaged mean bedtime was 23:31, and the mean wake time was 07:25. On weekends (n = 280 observations), participants slept longer (M = 9.93 hours, SD = 4.85 hours) and later, with a mean bedtime of 23:38 and wake time of 08:01. The average sleep duration represents the mean of each individual night's reported sleep period, which differs from the interval between overall average bedtime and wake time due to variability in nightly sleep behavior.

Linking Passive Data to Sleep Windows. Smartphone use covariates were derived from timestamped screenshots collected passively at approximately five-second intervals during active phone use via the Screenlife Capture app. Each EMA entry was linked to the sleep window reported for the previous night. Screenshots falling within the self-reported bedtime and wake time interval were used to compute two primary variables:

- Phone minutes during sleep window: calculated as the total number of screenshots in the window multiplied by five seconds and converted to minutes.
- Keyboard minutes during sleep window: the subset of the above duration in which a keyboard was detected on screen.

To account for general phone usage trends, we created matched control variables based on the 24-hour period preceding each EMA, excluding the reported sleep window:

- Phone minutes outside sleep window
- Keyboard minutes outside sleep window

© 2025 Jacobucci R et al. *JAMA Network Open*.

All four usage variables were standardized and decomposed into between-person and within-person components prior to multilevel modeling.

Study 3: Method

To examine hour-by-hour assocations of nighttime smartphone use, we constructed a complete hourly usage dataset spanning the nighttime window from 11:00 PM (23:00) to 8:00 AM (08:00), consistent with average sleep timing in the sample. For each participant and calendar day, **screenshot-derived phone activity** was aggregated into individual hours (specifically hours 23, 0, 1, 2, 3, 4, 5, 6, and 7), producing hourly variables for **phone use duration** and **keyboard use duration.**

A **complete data grid** was generated for each day on which the participant completed at least one EMA, creating one row for each hour in the nighttime window. If no screenshots were detected for a given hour, values of zero were assigned for both phone and keyboard use. These hourly usage metrics were then merged with the EMA dataset such that **each EMA response was linked to all nine hours of the preceding night,** creating a long data structure suitable for modeling time-dependent relationships.

To control for general usage trends, **prior day phone use** and **keyboard use** were calculated for the **daytime period (8:00 AM to 11:00 PM)** on the day prior to each EMA. All usage covariates and **subjective sleep quality** were z-scored and then decomposed into between-person and within-person components before inclusion in multilevel models.

For categorical analyses, the **hour of night** variable was grouped into three bins:

- **Late night:** 11:00 PM to 1:00 AM (hours 23:00–00:59)
- **Middle of the night**: 1:00 AM to 5:00 AM (01:00–04:59)
- **Early morning:** 5:00 AM to 8:00 AM (05:00–07:59)

Analyses focused on models testing **interactions between hour of night and smartphone use covariates** in relation to next-day suicide risk.

For fitting the spline models, we used the built-in function s() in brms to specify a smoothing term for the within-person longest night gap variable. To compare the fit of these models to the models with categorical representations of the night gap variable, we used the leave-one-out cross-validation criterion.

© 2025 Jacobucci R et al. *JAMA Network Open*.

eTable 1. Study 1 Variables Estimating Passive Suicidal Ideation (Categorical Gap Model)

Parameter	B	SE	95% CI	Rhat	Bulk ESS	Tail ESS
**Fixed Effects**						
Intercept[1]	0.89	0.31	[0.23, 1.47]	1.01	182	355
Intercept[2]	1.46	0.31	[0.80, 2.05]	1.01	184	440
Intercept[3]	3.02	0.31	[2.37, 3.63]	1.01	189	420
Intercept[4]	3.60	0.31	[2.93, 4.21]	1.01	191	424
Intercept[5]	4.69	0.32	[4.01, 5.32]	1.01	206	324
Intercept[6]	5.05	0.32	[4.37, 5.68]	1.01	208	360
Intercept[7]	5.75	0.33	[5.07, 6.38]	1.01	216	407
Intercept[8]	6.01	0.33	[5.32, 6.66]	1.01	220	428
Gap category < 4h	-0.05	0.14	[-0.32, 0.21]	1.00	1354	1595
Gap category 4-7h	0.35	0.11	[0.14, 0.55]	1.00	1341	1517
Gap category ≥ 9h	0.20	0.11	[-0.01, 0.40]	1.00	1630	1597
Between-person prior day row count	0.61	0.37	[-0.12, 1.33]	1.01	296	357
Within-person prior day row count	-0.06	0.05	[-0.15, 0.04]	1.00	1959	1354
Between-person subjective sleep	-0.92	0.27	[-1.44, -0.38]	1.01	244	489
**Random Effects**						
SD (Intercept)	2.27	0.24	[1.83, 2.81]	1.02	333	555

Note. All continuous covariates were standardized (z-scored). Reference category for gap is 7-9h. CI = credible interval; ESS = effective sample size. Intercepts represent the thresholds between adjacent response categories in the ordinal multilevel model.

© 2025 Jacobucci R et al. *JAMA Network Open*.

eTable 2. Study 1 Variables Estimating Active Suicidal Ideation (Categorical Gap Model)

Parameter	B	SE	95% CI	Rhat	Bulk ESS	Tail ESS
**Fixed Effects**						
Intercept[1]	0.92	0.28	[0.38, 1.45]	1.03	162	325
Intercept[2]	1.89	0.28	[1.33, 2.44]	1.03	165	319
Intercept[3]	3.48	0.29	[2.92, 4.03]	1.03	170	316
Intercept[4]	4.16	0.29	[3.58, 4.71]	1.03	172	332
Intercept[5]	5.23	0.30	[4.63, 5.81]	1.03	189	342
Intercept[6]	5.60	0.31	[5.01, 6.19]	1.02	194	374
Intercept[7]	6.41	0.32	[5.76, 7.04]	1.02	216	425
Intercept[8]	6.60	0.33	[5.92, 7.24]	1.02	226	460
Gap category < 4h	-0.15	0.14	[-0.42, 0.11]	1.00	1517	1478
Gap category 4-7h	0.29	0.10	[0.10, 0.49]	1.00	1426	1372
Gap category ≥ 9h	0.17	0.11	[-0.04, 0.40]	1.00	1757	1582
Between-person prior day row count	0.52	0.34	[-0.12, 1.23]	1.01	261	443
Within-person prior day row count	-0.08	0.05	[-0.18, 0.02]	1.00	2425	1470
Between-person subjective sleep	-0.92	0.23	[-1.39, -0.48]	1.02	165	457
**Random Effects**						
SD (Intercept)	2.10	0.22	[1.72, 2.61]	1.02	364	447

Note. All continuous covariates were standardized (z-scored). Reference category for gap is 7-9h. CI = credible interval; ESS = effective sample size. Intercepts represent the thresholds between adjacent response categories in the ordinal multilevel model.

© 2025 Jacobucci R et al. *JAMA Network Open*.

eTable 3. Study 1 Variables Estimating Suicide Planning (Categorical Gap Model)

Parameter	B	SE	95% CI	Rhat	Bulk ESS	Tail ESS
**Fixed Effects**						
Intercept	-3.07	0.32	[-3.74, -2.49]	1.03	320	696
Gap category < 4h	-0.23	0.22	[-0.65, 0.21]	1.00	2383	1677
Gap category 4-7h	0.10	0.16	[-0.20, 0.42]	1.00	2036	1658
Gap category ≥ 9h	0.18	0.16	[-0.13, 0.50]	1.00	2419	1612
Between-person prior day row count	0.24	0.38	[-0.52, 0.98]	1.00	473	788
Within-person prior day row count	0.08	0.08	[-0.09, 0.24]	1.00	3111	1563
Between-person subjective sleep	-0.57	0.28	[-1.13, -0.03]	1.01	411	735
**Random Effects**						
SD (Intercept)	2.13	0.25	[1.67, 2.67]	1.00	492	764

Note. All continuous covariates were standardized (z-scored). Reference category for gap is 7-9h. CI = credible interval; ESS = effective sample size.

© 2025 Jacobucci R et al. *JAMA Network Open*.

eTable 4. Study 2 Variables Estimating Passive Suicidal Ideation

Parameter	B	SE	95% CI	Rhat	Bulk ESS	Tail ESS
**Fixed Effects**						
Intercept[1]	0.92	0.33	[0.22, 1.54]	1.01	398	538
Intercept[2]	1.50	0.33	[0.81, 2.12]	1.01	402	546
Intercept[3]	3.04	0.33	[2.36, 3.69]	1.01	408	532
Intercept[4]	3.64	0.34	[2.96, 4.30]	1.01	418	501
Intercept[5]	4.77	0.34	[4.05, 5.45]	1.01	435	568
Intercept[6]	5.09	0.35	[4.36, 5.78]	1.00	434	495
Intercept[7]	5.94	0.36	[5.19, 6.65]	1.00	451	545
Intercept[8]	6.18	0.37	[5.41, 6.91]	1.00	465	601
Between-person sleep phone minutes	-0.34	1.04	[-2.38, 1.71]	1.01	489	647
Within-person sleep phone minutes	0.15	0.06	[0.04, 0.26]	1.00	1755	1412
Between-person sleep keyboard minutes	0.54	1.23	[-1.77, 2.95]	1.01	525	795
Within-person sleep keyboard minutes	-0.14	0.05	[-0.24, -0.04]	1.00	2160	1613
Between-person non-sleep phone minutes	0.17	0.57	[-0.84, 1.29]	1.01	415	584
Within-person non-sleep phone minutes	0.02	0.08	[-0.15, 0.18]	1.00	1483	1337
Between-person non-sleep keyboard minutes	0.66	0.74	[-0.84, 2.05]	1.00	472	559
Within-person non-sleep keyboard minutes	-0.16	0.07	[-0.29, -0.03]	1.00	1688	1566
Between-person subjective sleep	-0.96	0.31	[-1.57, -0.34]	1.01	418	809
**Random Effects**						
SD (Intercept)	2.48	0.30	[1.94, 3.13]	1.01	347	723

Note. All continuous covariates were standardized (z-scored). CI = credible interval; ESS = effective sample size. Intercepts represent the thresholds between adjacent response categories in the ordinal multilevel model.

© 2025 Jacobucci R et al. *JAMA Network Open*.

eTable 5. Study 2 Variables Estimating Active Suicidal Ideation

Parameter	B	SE	95% CI	Rhat	Bulk ESS	Tail ESS
**Fixed Effects**						
Intercept[1]	0.95	0.32	[0.34, 1.58]	1.01	293	634
Intercept[2]	1.97	0.32	[1.35, 2.59]	1.01	295	634
Intercept[3]	3.71	0.32	[3.10, 4.35]	1.01	309	681
Intercept[4]	4.42	0.33	[3.80, 5.08]	1.01	327	735
Intercept[5]	5.53	0.34	[4.88, 6.23]	1.01	327	722
Intercept[6]	5.89	0.35	[5.24, 6.60]	1.01	348	776
Intercept[7]	6.66	0.37	[5.94, 7.40]	1.01	393	653
Intercept[8]	6.89	0.38	[6.15, 7.62]	1.01	417	696
Between-person sleep phone minutes	0.50	0.94	[-1.36, 2.41]	1.01	403	710
Within-person sleep phone minutes	0.06	0.05	[-0.05, 0.16]	1.00	1682	1314
Between-person sleep keyboard minutes	-0.18	1.11	[-2.39, 2.07]	1.01	374	682
Within-person sleep keyboard minutes	-0.13	0.06	[-0.24, -0.02]	1.00	2084	1349
Between-person non-sleep phone minutes	0.00	0.55	[-1.03, 1.04]	1.00	367	585
Within-person non-sleep phone minutes	-0.08	0.08	[-0.24, 0.07]	1.00	1560	1415
Between-person non-sleep keyboard minutes	0.74	0.67	[-0.61, 2.06]	1.00	365	561
Within-person non-sleep keyboard minutes	-0.12	0.06	[-0.24, -0.001]	1.00	1738	1270
Between-person subjective sleep	-1.02	0.29	[-1.59, -0.44]	1.01	423	824
**Random Effects**						
SD (Intercept)	2.33	0.27	[1.89, 2.92]	1.01	442	568

Note. All continuous covariates were standardized (z-scored). CI = credible interval; ESS = effective sample size. Intercepts represent the thresholds between adjacent response categories in the ordinal multilevel model.

© 2025 Jacobucci R et al. *JAMA Network Open*.

eTable 6. Study 2 Variables Estimating Suicide Planning (Binary)

Parameter	B	SE	95% CI	Rhat	Bulk ESS	Tail ESS
**Fixed Effects**						
Intercept	-3.38	0.41	[-4.26, -2.62]	1.01	516	733
Between-person sleep phone minutes	1.65	1.24	[-0.68, 4.26]	1.01	533	762
Within-person sleep phone minutes	0.13	0.09	[-0.04, 0.31]	1.00	2136	1436
Between-person sleep keyboard minutes	-2.02	1.45	[-5.05, 0.72]	1.01	612	888
Within-person sleep keyboard minutes	-0.05	0.15	[-0.35, 0.22]	1.00	2022	1745
Between-person non-sleep phone minutes	-0.26	0.64	[-1.56, 0.98]	1.00	603	695
Within-person non-sleep phone minutes	-0.21	0.12	[-0.46, 0.03]	1.00	2340	1430
Between-person non-sleep keyboard minutes	0.31	0.81	[-1.28, 1.90]	1.00	694	1044
Within-person non-sleep keyboard minutes	0.13	0.11	[-0.10, 0.34]	1.00	2324	1647
Between-person subjective sleep	-0.73	0.37	[-1.42, -0.05]	1.01	526	891
**Random Effects**						
SD (Intercept)	2.67	0.41	[1.99, 3.60]	1.00	484	1074

Note. All continuous covariates were standardized (z-scored). CI = credible interval; ESS = effective sample size.

© 2025 Jacobucci R et al. *JAMA Network Open*.

eTable 7. Study 3 Variables Estimating Passive Suicidal Ideation

Parameter	B	SE	95% CI	Rhat	Bulk ESS	Tail ESS
**Fixed Effects**						
Intercept[1]	0.98	0.33	[0.33, 1.64]	1.00	417	579
Intercept[2]	1.56	0.33	[0.91, 2.21]	1.00	417	604
Intercept[3]	3.13	0.33	[2.49, 3.78]	1.00	419	576
Intercept[4]	3.72	0.33	[3.08, 4.37]	1.00	421	552
Intercept[5]	4.82	0.33	[4.19, 5.47]	1.00	422	561
Intercept[6]	5.19	0.33	[4.54, 5.82]	1.00	425	600
Intercept[7]	5.88	0.33	[5.23, 6.53]	1.00	431	590
Intercept[8]	6.13	0.33	[5.47, 6.79]	1.00	430	624
**Main Effects**						
Between-person hourly night phone minutes	1.61	1.42	[-1.12, 4.12]	1.01	564	734
Within-person hourly night phone minutes	0.13	0.03	[0.08, 0.18]	1.01	1331	1053
Between-person hourly night keyboard minutes	0.86	1.77	[-2.56, 4.39]	1.01	722	744
Within-person hourly night keyboard minutes	-0.03	0.02	[-0.07, 0.02]	1.00	1281	1126
Between-person previous day phone minutes	-1.12	0.86	[-2.77, 0.61]	1.01	502	483
Within-person previous day phone minutes	0.08	0.02	[0.04, 0.12]	1.00	1550	909
Between-person previous day keyboard minutes	1.23	0.84	[-0.49, 2.89]	1.01	568	603
Within-person previous day keyboard minutes	-0.23	0.02	[-0.27, -0.19]	1.00	1533	900
Between-person subjective sleep	-1.03	0.33	[-1.65, -0.40]	1.00	513	537
Late night (11pm-1am)	0.04	0.03	[-0.03, 0.10]	1.00	1734	901
Middle night (1am-5am)	0.03	0.03	[-0.04, 0.10]	1.00	1710	919
**Interaction Effects**						
WP phone minutes × Late night	-0.10	0.03	[-0.16, -0.03]	1.00	1345	1097
WP phone minutes × Middle night	-0.20	0.04	[-0.27, -0.12]	1.00	1381	1064
WP keyboard minutes × Late night	-0.07	0.03	[-0.13, -0.02]	1.00	1412	1125
WP keyboard minutes × Middle night	0.07	0.03	[0.00, 0.13]	1.00	1329	1169

© 2025 Jacobucci R et al. *JAMA Network Open*.

Parameter	B	SE	95% CI	Rhat	Bulk ESS	Tail ESS
**Random Effects**						
SD (Intercept)	2.70	0.28	[2.20, 3.29]	1.01	403	538

Note. All continuous covariates were standardized (z-scored). Reference category for time of night is early night (9pm-11pm). WP = within-person; CI = credible interval; ESS = effective sample size. Intercepts represent the thresholds between adjacent response categories in the ordinal multilevel model.

© 2025 Jacobucci R et al. *JAMA Network Open*.

eTable 8. Study 3 Variables Estimating Active Suicidal Ideation

Parameter	B	SE	95% CI	Rhat	Bulk ESS	Tail ESS
**Fixed Effects**						
Intercept[1]	1.02	0.30	[0.41, 1.61]	1.02	444	478
Intercept[2]	2.00	0.30	[1.40, 2.58]	1.02	442	484
Intercept[3]	3.62	0.30	[3.00, 4.19]	1.02	448	548
Intercept[4]	4.30	0.31	[3.68, 4.88]	1.02	448	513
Intercept[5]	5.37	0.31	[4.74, 5.95]	1.02	455	536
Intercept[6]	5.74	0.31	[5.10, 6.32]	1.02	455	582
Intercept[7]	6.54	0.31	[5.91, 7.13]	1.02	479	575
Intercept[8]	6.72	0.31	[6.08, 7.29]	1.02	482	620
**Main Effects**						
Between-person hourly night phone minutes	2.27	1.18	[0.03, 4.52]	1.01	579	651
Within-person hourly night phone minutes	0.10	0.03	[0.05, 0.15]	1.00	1250	1063
Between-person hourly night keyboard minutes	0.43	1.55	[-2.63, 3.52]	1.01	634	570
Within-person hourly night keyboard minutes	0.02	0.02	[-0.02, 0.07]	1.00	1243	798
Between-person previous day phone minutes	-1.73	0.74	[-3.18, -0.38]	1.01	623	775
Within-person previous day phone minutes	0.08	0.02	[0.04, 0.12]	1.00	1692	1109
Between-person previous day keyboard minutes	1.57	0.74	[0.14, 2.98]	1.00	494	666
Within-person previous day keyboard minutes	-0.23	0.02	[-0.27, -0.19]	1.00	1529	956
Between-person subjective sleep	-1.08	0.28	[-1.67, -0.54]	1.00	512	548
Late night (11pm-1am)	0.04	0.03	[-0.03, 0.10]	1.00	1290	884
Middle night (1am-5am)	0.02	0.03	[-0.04, 0.09]	1.00	1521	1174
**Interaction Effects**						
WP phone minutes × Late night	-0.14	0.03	[-0.20, -0.08]	1.00	1468	1088
WP phone minutes × Middle night	-0.23	0.04	[-0.30, -0.15]	1.01	1198	886
WP keyboard minutes × Late night	-0.05	0.03	[-0.11, 0.00]	1.00	1314	945
WP keyboard minutes × Middle night	-0.01	0.04	[-0.08, 0.06]	1.01	1285	911

© 2025 Jacobucci R et al. *JAMA Network Open*.

Parameter	B	SE	95% CI	Rhat	Bulk ESS	Tail ESS
**Random Effects**						
SD (Intercept)	2.34	0.25	[1.89, 2.88]	1.01	360	590

Note. All continuous covariates were standardized (z-scored). Reference category for time of night is early night (9pm-11pm). WP = within-person; CI = credible interval; ESS = effective sample size. Intercepts represent the thresholds between adjacent response categories in the ordinal multilevel model.

© 2025 Jacobucci R et al. *JAMA Network Open*.

eTable 9. Study 3 Variables Predicting Suicidal Planning (Binary)

Parameter	B	SE	95% CI	Rhat	Bulk ESS	Tail ESS
**Fixed Effects**						
Intercept	-3.58	0.38	[-4.38, -2.90]	1.00	359	534
**Main Effects**						
Between-person hourly night phone minutes	3.26	1.57	[0.04, 6.21]	1.01	362	481
Within-person hourly night phone minutes	0.08	0.04	[-0.01, 0.16]	1.01	1022	835
Between-person hourly night keyboard minutes	-2.93	2.04	[-7.03, 0.87]	1.01	381	598
Within-person hourly night keyboard minutes	0.01	0.04	[-0.08, 0.09]	1.00	960	963
Between-person previous day phone minutes	-1.25	0.94	[-3.13, 0.58]	1.01	388	565
Within-person previous day phone minutes	0.08	0.03	[0.02, 0.15]	1.00	1524	1095
Between-person previous day keyboard minutes	0.78	0.99	[-1.11, 2.72]	1.01	394	624
Within-person previous day keyboard minutes	-0.06	0.03	[-0.13, -0.002]	1.00	1874	1052
Between-person subjective sleep	-0.70	0.35	[-1.41, -0.09]	1.01	313	464
Late night (11pm-1am)	0.03	0.05	[-0.08, 0.13]	1.00	1329	887
Middle night (1am-5am)	0.01	0.06	[-0.10, 0.12]	1.00	1309	1021
**Interaction Effects**						
WP phone minutes × Late night	-0.15	0.05	[-0.25, -0.05]	1.00	1138	1038
WP phone minutes × Middle night	-0.18	0.06	[-0.29, -0.07]	1.01	1218	991
WP keyboard minutes × Late night	0.02	0.06	[-0.09, 0.12]	1.00	1014	842
WP keyboard minutes × Middle night	0.11	0.06	[0.00, 0.23]	1.00	1164	984
**Random Effects**						
SD (Intercept)	2.90	0.37	[2.30, 3.75]	1.01	227	678

Note. All continuous covariates were standardized (z-scored). Reference category for time of night is early night (9pm-11pm). WP = within-person; CI = credible interval; ESS = effective sample size.

© 2025 Jacobucci R et al. *JAMA Network Open*.

eFigure 1. Association Between Maximum Phone-Free Gap and Self-Reported Sleep Duration

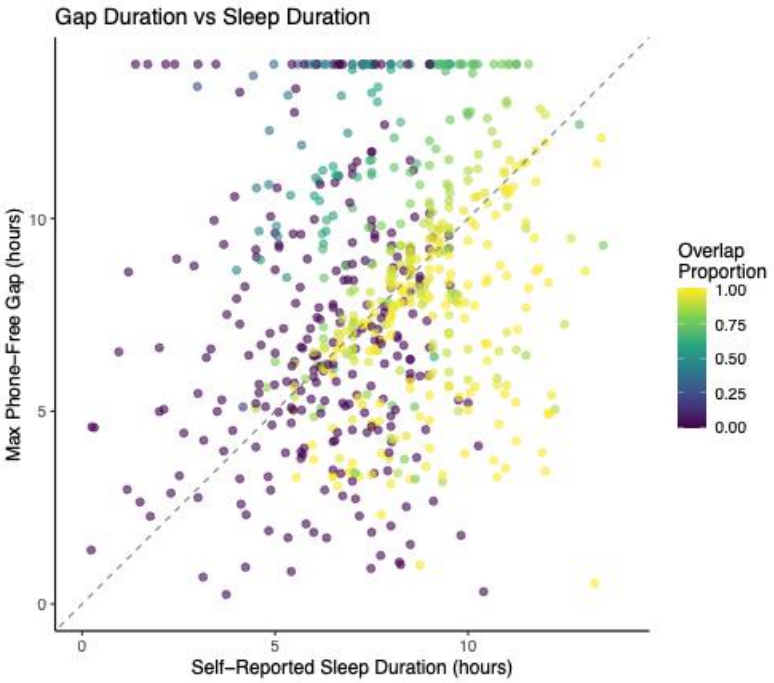

Note: Scatter plot comparing maximum phone-free gap duration (y-axis) to self-reported sleep duration (x-axis) for all person-days with available data. Each point represents one person-day, with color indicating the proportion of the gap that overlapped with self-reported sleep (0 = no overlap, 1 = complete overlap). The diagonal dashed line represents perfect agreement where gap duration equals sleep duration. Points above the line indicate phone-free gaps longer than self-reported sleep; points below indicate gaps shorter than self-reported sleep. The correlation between both metrics was 0.101.

eFigure 2. Random Effects of Hour of Night in Estimating Passive Suicidal Ideation

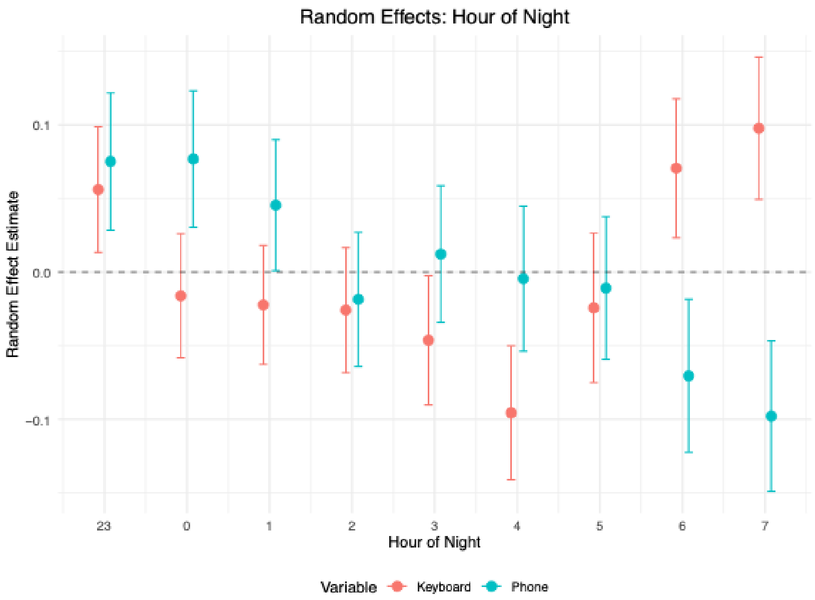

Note: Random effects estimates from multilevel models examining the association between hour of night and smartphone use behaviors. Error bars represent standard errors of the random effect estimates. Phone usage refers to smartphone screen time (wp_hourly_night_phone_min_z); keyboard usage refers to computer keyboard activity (wp_hourly_night_keyb_min_z). Hours are displayed in chronological order from 23:00 (11 PM) through 07:00 (7 AM).

© 2025 Jacobucci R et al. *JAMA Network Open*.

eFigure 3. Random Effects of Hour of Night in Estimating Active Suicidal Ideation

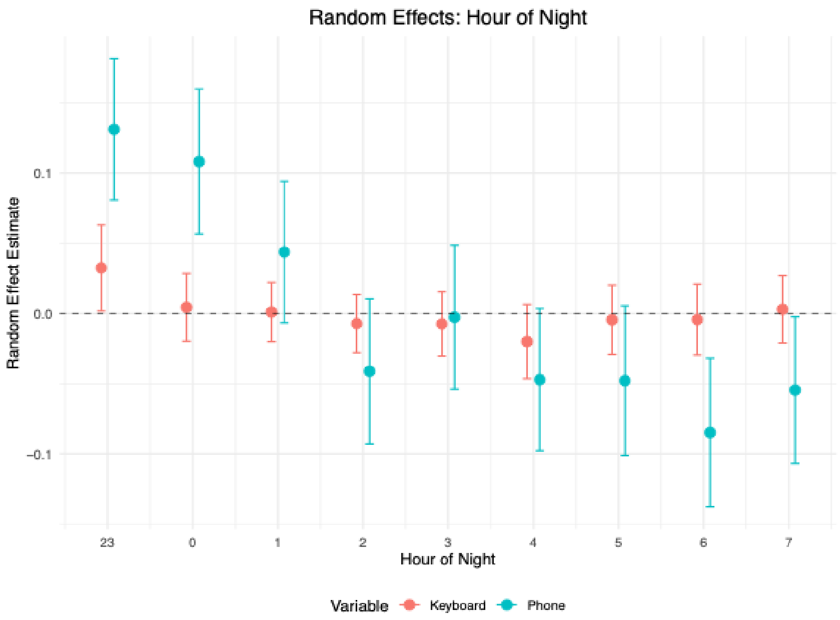

Note: Random effects estimates from multilevel models examining the association between hour of night and smartphone use behaviors. Error bars represent standard errors of the random effect estimates. Phone usage refers to smartphone screen time (wp_hourly_night_phone_min_z); keyboard usage refers to computer keyboard activity (wp_hourly_night_keyb_min_z). Hours are displayed in chronological order from 23:00 (11 PM) through 07:00 (7 AM).

© 2025 Jacobucci R et al. *JAMA Network Open*.

eFigure 4. Random Effects of Hour of Night in Estimating Suicide Planning

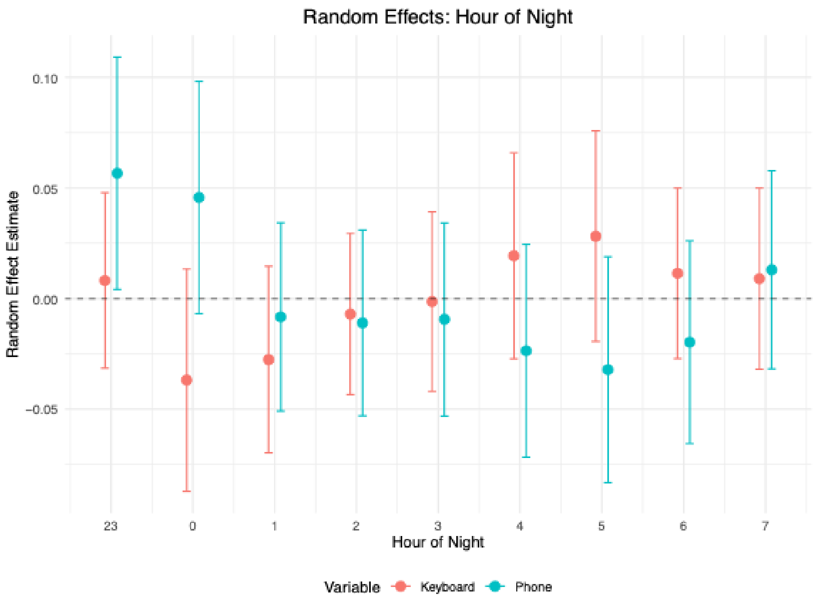

Note: Random effects estimates from multilevel models examining the association between hour of night and smartphone use behaviors. Error bars represent standard errors of the random effect estimates. Phone usage refers to smartphone screen time (wp_hourly_night_phone_min_z); keyboard usage refers to computer keyboard activity (wp_hourly_night_keyb_min_z). Hours are displayed in chronological order from 23:00 (11 PM) through 07:00 (7 AM).

© 2025 Jacobucci R et al. *JAMA Network Open*.

eFigure 5. Distribution of *Maximum Nighttime Phone-Free Gap*

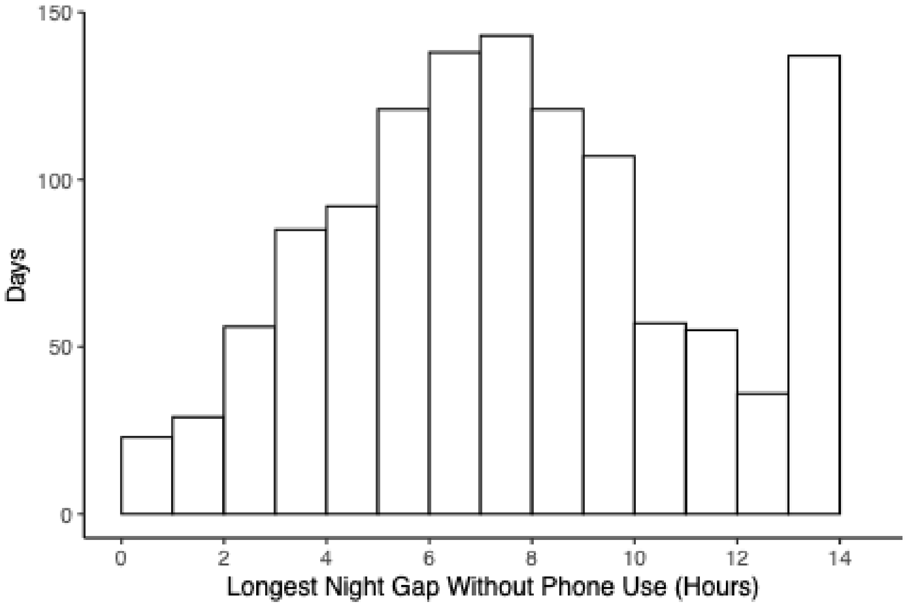

© 2025 Jacobucci R et al. *JAMA Network Open*.

eFigure 6. Interaction for Within-Person Hourly Nighttime Smartphone Use Estimating Passive Suicidal Ideation

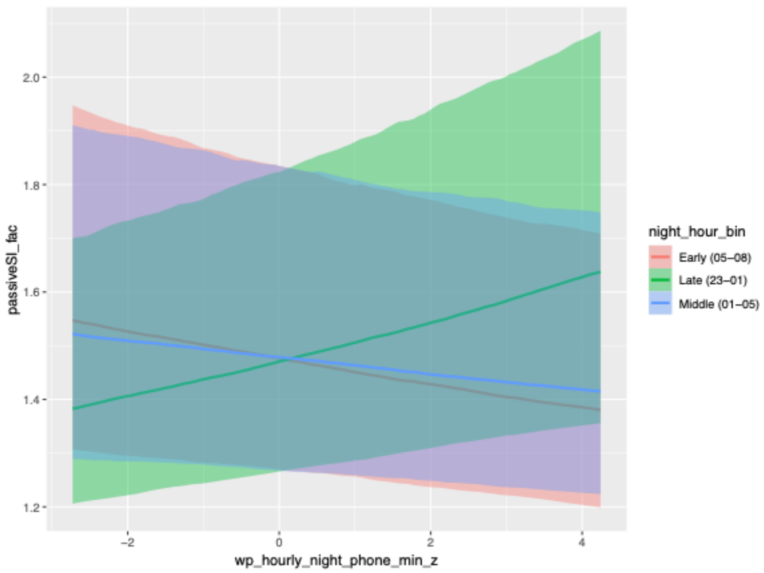

Note: The association between higher within-person nighttime smartphone use and passive suicidal ideation levels was more positive during the Late night bin compared to the Early morning bin.

© 2025 Jacobucci R et al. *JAMA Network Open*.

eFigure 7. Interaction for Within-Person Hourly Nighttime Keyboard Use Estimating Passive Suicidal Ideation

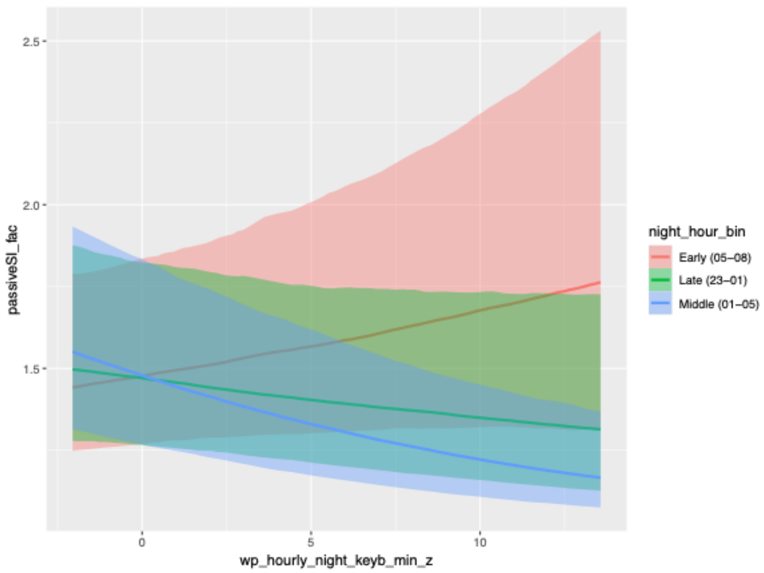

Note: The association between higher within-person nighttime keyboard use and passive suicidal ideation was more negative during the Middle bin compared to the Early morning.

© 2025 Jacobucci R et al. *JAMA Network Open*.

eFigure 8. Interaction for Within-Person Hourly Nighttime Smartphone Use Estimating Active Suicidal Ideation

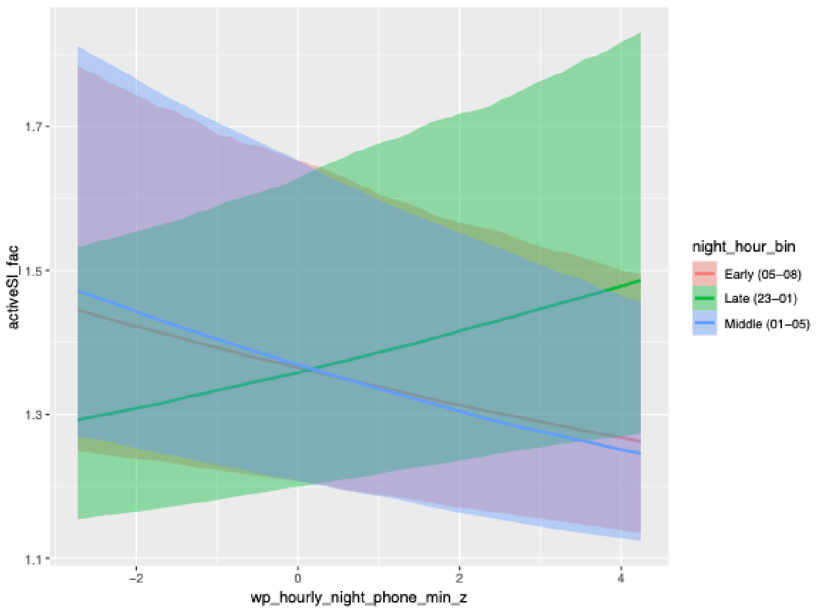

Note: The association between higher within-person nighttime smartphone use and active suicidal ideation levels was more positive during the Late night bin compared to the Early morning bin.

© 2025 Jacobucci R et al. *JAMA Network Open*.

eFigure 9. Interaction for Within-Person Hourly Nighttime Smartphone Use Estimating Suicide Planning

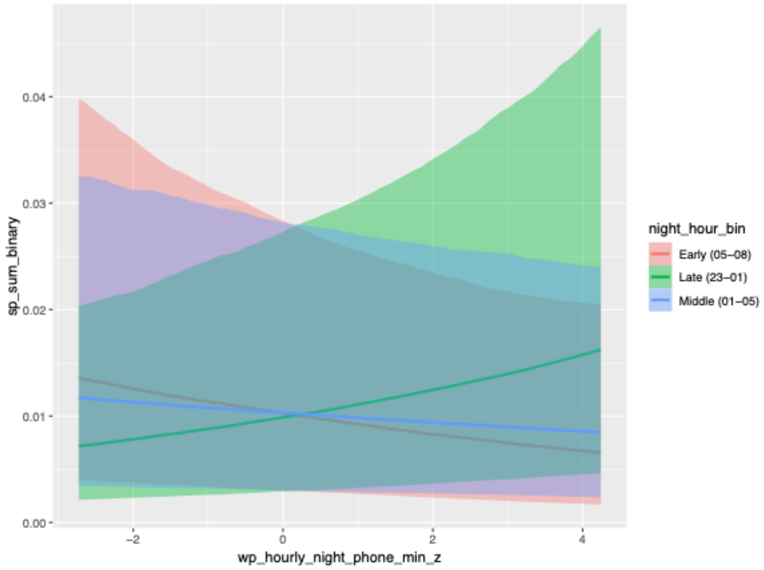

Note: The association between within-person nighttime smartphone use and the odds of suicidal planning was more positive during the Late night bin

© 2025 Jacobucci R et al. *JAMA Network Open*.
