# Supplementary material for: Passive vs Active Nighttime Smartphone Use as Markers of Next-Day Suicide Risk
Source: JAMA Netw Open. 2025 Nov 11;8(11):e2542675. doi: 10.1001/jamanetworkopen.2025.42675 (PMC12606377; doi:10.1001/jamanetworkopen.2025.42675)
Supplement: Supplement 2. — Data Sharing Statement [file jamanetwopen-e2542675-s002.pdf]

Data Sharing Statement

Jacobucci. Passive vs Active Nighttime Smartphone Use as Markers of Next-Day Suicide Risk. *JAMA Netw Open*. Published November 11, 2025. doi:10.1001/jamanetworkopen.2025.42675

Data
Data available: No
